# Study protocol for impact of visual inhaler technique instructions on short-term outcomes in hospitalized patients with acute exacerbation of chronic obstructive pulmonary disease

**DOI:** 10.3389/fmed.2025.1735550

**Published:** 2026-01-09

**Authors:** Siyuan Wang, Yuxuan Ji, Beiyao Gao, Peijian Wang, Shiwei Qumu, Fen Dong, Yuanwen Wang, Ting Yang, Shan Jiang

**Affiliations:** 1 Department of Rehabilitation Medicine, China-Japan Friendship Hospital, Beijing, China; 2 Department of Rehabilitation Medicine, Xiyuan Hospital, China Academy of Chinese Medical Sciences, Beijing, China; 3 National Center for Respiratory Diseases, Beijing, China; 4 Institute of Respiratory Medicine, Chinese Academy of Medical Sciences, Beijing, China; 5 National Clinical Research Center for Respiratory Diseases, Beijing, China; 6 Department of Pulmonary and Critical Care Medicine, Center of Respiratory Medicine, China-Japan Friendship Hospital, Beijing, China; 7 Department of Clinical Research and Data Management, Center of Respiratory Medicine, China Japan Friendship Hospital, Beijing, China; 8 Department of Tuberculosis, Anhui Chest Hospital, Hefei, Anhui, China

**Keywords:** acute exacerbation of COPD, inhaler technique (IT), visual instructions, RCT—randomized controlled trial, study protocol

## Abstract

**Background:**

Acute exacerbations (AEs) are key events in the progression of chronic obstructive pulmonary disease (COPD), accelerating the decline in lung function and increasing mortality considerably. Inhaler therapy is essential for COPD management; however, incorrect use of inhalation techniques can reduce drug efficacy and increase AE frequency. To optimize the effectiveness of inhaler use, we aim to conduct a randomized controlled trial, in which short-term visual instructions for inhalation use are provided to hospitalized patients with AE of COPD (AECOPD) to reduce AE frequency and severity within 12 weeks.

**Methods:**

This will be a single-center, randomized controlled trial including hospitalized patients with AECOPD who meet the inclusion criteria at the China–Japan Friendship Hospital. The patients will be randomly assigned to a visual inhaler technique instruction (VIT; experimental) or traditional inhaler technique education (TIE; control) group. During hospitalization, the TIE group will receive two standardized TIE sessions conducted by a respiratory nurse. In contrast, the VIT group will receive two VIT sessions under a physical therapist's supervision. The primary outcome will be AE frequency within 12 weeks after discharge. Secondary outcomes include all-cause mortality and each AE's severity (EXACT-Pro score) over 12 post-discharge weeks. Secondary outcomes will also include lung function, physical fitness (6-min walk test score, grip strength, and maximum voluntary contraction of knee extension), respiratory muscle strength (maximal inspiratory and expiratory pressures), dyspnea severity (modified Medical Research Council scale score), inhaler medication adherence, and quality of life (COPD Assessment Test score) at week 12 after discharge.

**Discussion:**

We aim to address the issues related to inhaler technique education that are often overlooked in clinical practice. Our findings may provide evidence for clinical practice and enable further optimization of inhaler technique education management strategies for patients with AECOPD.

## 1 Background

Chronic obstructive pulmonary disease (COPD) is a progressive disorder characterized by persistent inflammation of the airways, alveoli, and microvasculature, primarily manifested by irreversible airflow limitation (1). Acute exacerbations (AEs) are major negative events occurring in the course of COPD. Clinical observations have demonstrated that patients experiencing frequent AEs of COPD (AECOPD) have a significantly faster decline in lung function indicators, such as forced expiratory volume in 1 s (FEV1) and peak expiratory flow (PEF), compared to those with a stable disease (2). After the occurrence of an AE requiring hospitalization, the risk of mortality in the short term increases significantly (3, 4). Even after remission, patients with AECOPD have a high recurrence risk. In patients with AECOPD, the rate of AE recurrence within 90 postdischarge days can be as high as 48.9% (5). The more frequent the recurrences, the higher the mortality rate (4). Therefore, reducing AE recurrence is essential for improving the quality of life and reducing mortality, healthcare visit frequency, and medical costs among affected patients with COPD (6, 7). According to the 2024 report of the Global Initiative for Chronic Obstructive Lung Disease (GOLD), inhaler therapy is the preferred treatment for stable COPD patients, and correct use of inhaled medications can reduce the mortality risk in patients with a history of frequent AEs, severe AEs, or both significantly (8, 9).

During inhalation therapy, the drug delivery and deposition process involves the medication traveling through the oral cavity and pharynx, followed by its entry into the trachea, bronchi, and small airways, and ultimately its arrival and deposition into the alveoli and small airways. Here, the patient's mastery of the correct inhalation technique mainly influences the effectiveness of drug delivery and deposition. In particular, this effectiveness is closely related to not only the appropriate maintenance of inspiratory flow rate but also the proper execution of inhalation techniques.

Numerous studies have confirmed that an improper inspiratory flow rate—too low or too high—can hinder drug delivery (3, 10). The most commonly used parameter for assessing the inhalation technique is peak inspiratory flow rate (PIFr); it is the maximum flow rate generated by a patient during inhalation to overcome the internal resistance of an inhaler (6). Different types of inhalers, such as pressurized metered dose inhaler (pMDI), dry powder inhaler (DPI), and soft mist inhaler (SMI), have varying PIFr requirements because of varied drug particle release mechanisms and device resistance. The optimal inspiratory flow rate for DPIs is approximately 60 L/min, which generates turbulence inside the device, facilitating the dispersion of the powder into fine particles that can be carried into the airways via airflow (11). In contrast, pMDIs and SMIs do not rely on the patient's active inhalation to generate turbulence for drug particle release and thus have an optimal inspiratory flow rate of about 30 L/min (12). If a patient's measured PIFr is suboptimal, it may limit medication delivery to the lower airways, thereby reducing efficacy (13). In addition to inspiratory flow rate, proper inhalation techniques are crucial for drug deposition. Adequate exhalation before inhalation minimizes residual volume, increasing the volume of medicated air inhaled and enhancing drug delivery to small airways. Furthermore, prolonging breath-holding after inhalation facilitates the deposition of fine drug particles in small airways via Brownian motion, reducing the proportion of exhaled suspended particles (14).

Many recent studies have considered inspiratory flow rate to be an independent evaluation parameter. However, inhaler device preparation, exhalation before inhalation, and breath-holding after inhalation are the main components of the inhalation technique, which are crucial for assessing the completeness and effectiveness of drug delivery. Most studies have primarily focused on individualized inhalation flow rate guidance based on PIFr measurements; however, studies combining PIFr assessment with a comprehensive inhalation technique training remain limited. Despite the importance of using the correct inhalation technique, inhaler technique education remains insufficient. According to the 2024 GOLD guidelines, physicians must combine explanation and demonstration to ensure understanding among their patients and verify their mastery of the technique by encouraging them to perform it onsite. However, 36%−67% of physicians cannot fully or accurately describe or master the key steps of inhaler device use (15). Moreover, research evaluating inhaler devices and training tools remains scant (16–18). In summary, inhalation technique evaluation and education can be considerably challenging and warrant improvement.

We therefore plan to conduct a randomized controlled trial comparing the effects of short-term visual inhaler technique instruction (VIT) and traditional inhaler technique education (TIE) before discharge in hospitalized patients with AECOPD. In particular, we will assess how the two interventions affect the rate of AEs within 12 weeks after discharge. Our objective is to collect data and establish clinical pathways for inhalation technique assessment and education.

## 2 Methods and analysis

### 2.1 Study design

This will be a single-center, prospective, and parallel randomized controlled trial. Participants will be randomly assigned to a VIT or TIE group for corresponding interventions and follow-up. The study period will begin 1 week before discharge and include a 12-week follow-up after discharge. Outcomes will be measured at baseline (V0), after instruction (V1), before discharge (V2), and at week 12 after discharge (V3).

**Figure 1:**
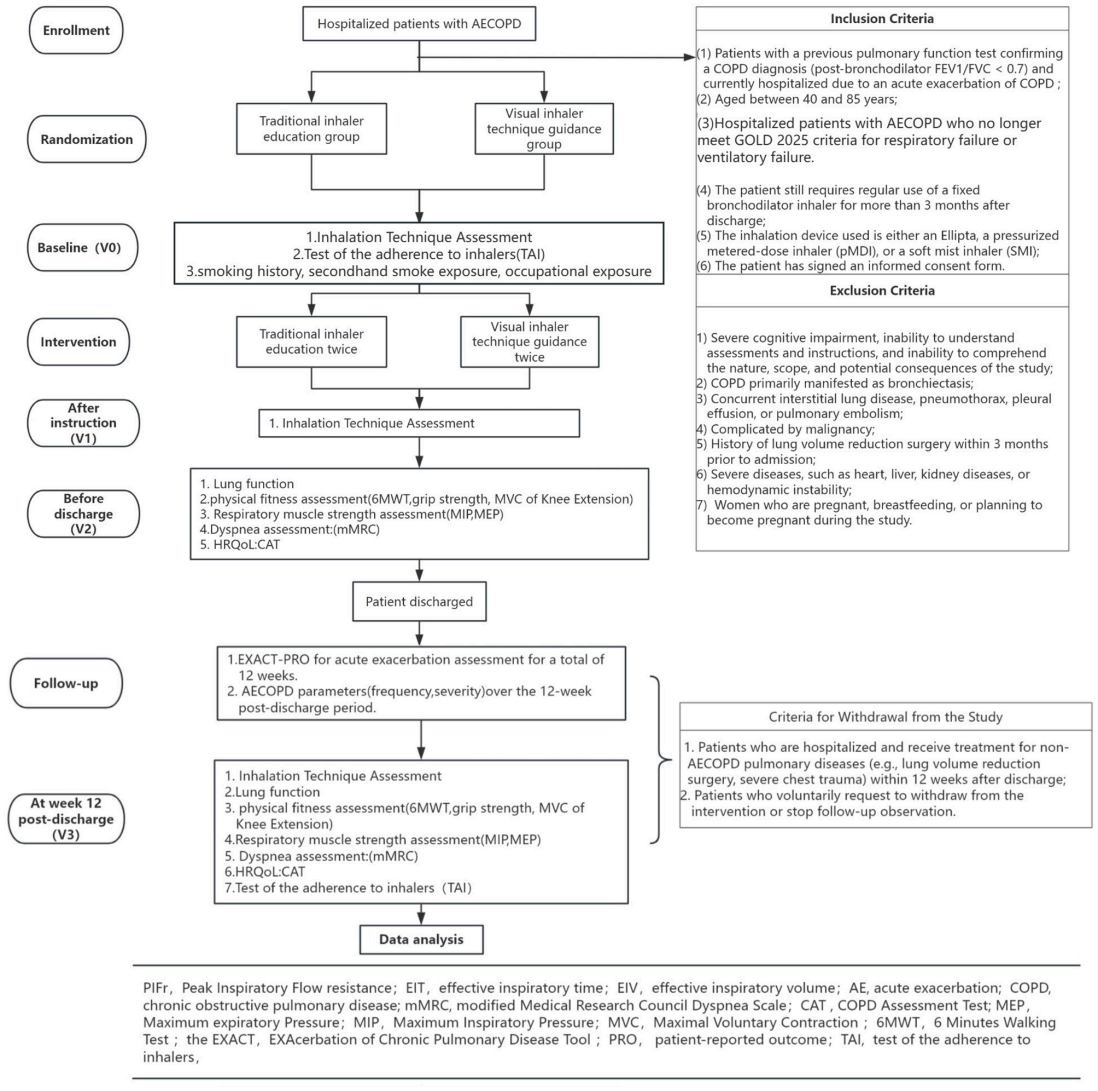
Technical flowchart of the present study.

This trial is registered at ClinicalTrials.gov (registration number: ChiCRT2500097816) on 26/02/2025. The study protocol is reported according to the Standard Protocol Items: Recommendations for Intervention Trials 2013 (19) guidelines, and the intervention procedures are described according to the Consolidated Standards of Reporting Trials (CONSORT) 2025 Checklists. Figure 1 illustrates the CONSORT flow diagram for this study, and Table 1 summarizes the study schedule and assessments.

**Table 1:** Summary of study schedule.

**Schedule of assessments and measurements across study time points**	**Enrollment**	**Baseline (V0)**	**After instruction (V1)**	**Before discharge (V2)**	**Follow-up**	**At week 12 post-discharge (V3)**
**Eligibility screening**						
Informed consent	✓					
Randomization		✓				
Allocation		✓				
**Interventions**						
VIT group		✓				
TIE group		✓				
**Inhalation technique assessment**						
PIFr		✓	✓			✓
EIT		✓	✓			✓
EIV		✓	✓			✓
IIFSs:		✓	✓			✓
IOCL		✓	✓			✓
**Pulmonary function**						
FVC & FVC%				✓		✓
FEV1 & FEV1%				✓		✓
FEV1/FVC				✓		✓
**Respiratory muscle strength assessment**						
MIP				✓		✓
MEP				✓		✓
**Assessment of physical function**						
Grip strength				✓		✓
MVC of Knee extension				✓		✓
6MWT				✓		✓
**Questionnaire**						
mMRC				✓		✓
CAT				✓		✓
TAI		✓				✓
EXACT-PRO					✓ (daily)	
Frequency and severity of AE					✓ (weekly)	

PIFr, peak inspiratory flow resistance; EIT, effective inspiratory time; EIV, effective inspiratory volume; IIFSC, integrated inspiratory flow system score; IOCL, inhalation operation chick list; MIP, maximum inspiratory pressure, MEP, maximum expiratory pressure, MVC, maximal voluntary contraction; 6MWT, 6 minutes walking test; mMRC, modified medical research council dyspnea scale; CAT, COPD assessment test; TAI, test of the adherence to inhalers; EXACT-PRO, exacerbation of chronic pulmonary disease tool-patient reported outcome; AE, acute exacerbations.

### 2.2 Study population and recruitment

All patients will undergo initial screening by physicians in the Department of Respiratory and Critical Care Medicine to confirm AECOPD diagnoses. Independent researchers will then conduct a secondary screening according to inclusion and exclusion criteria (Table 2) to ensure that only eligible patients are included. Patients or their legal guardians will be required to provide informed consent to participate.

**Table 2:** Inclusion, exclusion, and withdrawal criteria.

**Inclusion criteria**	**Exclusion criteria**	**Withdrawal criteria**
(1) A previous pulmonary function test confirming a COPD diagnosis (postbronchodilator FEV1/FVC < 0.7) and currently hospitalization due to AECOPD	(1) Severe cognitive impairment; inability to understand assessments or instructions; and inability to comprehend the study's nature, scope, and potential consequences	(1) Hospitalization and receipt of treatment for non-AECOPD pulmonary diseases (e.g., lung volume reduction surgery, severe chest trauma) within 12 weeks after discharge
(2) Age = 40–85 years	(2) Bronchiectasis as the primary COPD manifestation	(2) Voluntary request to withdraw from the intervention or terminate follow-up observations
(3) Hospitalized patients with AECOPD who no longer meet GOLD 2025 criteria for respiratory failure or ventilatory failure.	(3) Complication due to malignancy	
(4) Continual, regular use of a fixed bronchodilator inhaler for >3 months after discharge	(4) A history of lung volume reduction surgery within 3 months before admission;	
(5) Use of an Ellipta, pMDI, or soft mist inhaler (SMI) as the inhalation device	(5) Severe diseases, such as heart, liver, kidney diseases, or hemodynamic instability	
(6) Provision of written informed consent	(6) Currently pregnant, breastfeeding, or planning to become pregnant during the study	

### 2.3 Randomization, allocation concealment, and blinding

All patients will be randomly allocated at a 1:1 ratio to either the VIT or TIE group by using a random number table generated by an independent statistical analyst with SPSS. In accordance with the 2025 WHO age classification, participants will be stratified into two age groups (40–65 years and 66–85 years) before randomization to ensure balanced distribution across intervention arms. The assignment details will then be sealed in opaque envelopes. After each participant provides written informed consent and completes their baseline assessments, the study coordinator will open the envelope to reveal the participant's group allocation.

Because of the nature of the intervention, neither the participants nor the instructors will be blinded; nevertheless, the outcome assessors and statistical analysts will remain blinded to group assignments.

### 2.4 Interventions

All patients will receive two inhaler usage instruction sessions during hospitalization and will be followed up weekly via telephone over 12 weeks after discharge. Researchers blinded to the group assignments will implement this measure. It will ensure regular use of inhalation medications in both groups during the study period and enable recording of any possible AE events and their severity.

#### 2.4.1 VIT group

VIT will be conducted based on the patient's inhaler type. The training will be performed on the basis of the Visualized Inhalation Technique Training System (OHM-50, ORANGER DTx, Tianjin, China), which our research team has codeveloped. This system provides simulated inhalation training with visualized PIFr and inhalation duration (hereinafter, “visualized training”) and simulated inhalation training using a medication-free canister equipped with a sensor (hereinafter, “sensor training”).

In visualized training, patients will observe real-time inspiratory flow curves during inhalation and compare them with a standard reference chart. The therapist will provide instruction based on patient performance until the patient achieves the required inspiratory flow rate over three consecutive attempts. Each training session will allow for ≤ 10 attempts per day.

Next, each patient will undergo sensor training, using a medication-free inhaler canister equipped with an inhalation sensor for simulation. This sensor can detect four types of errors:

- E1: No inhalation
- E2: Hand-breath coordination issues
- E3: Insufficient inhalation duration
- E4: Comprehensive error (i.e., at least two of the aforementioned errors)

The therapist will also instruct patients to perform a breath-hold maneuver after inhalation is completed. On the basis of the observed errors and sensor feedback, the therapist will provide targeted guidance. If necessary, they will demonstrate the accurate technique until the patient successfully completes three consecutive correct inhalation attempts. Each training session will allow for ≤ 10 attempts per day.

#### 2.4.2 TIE group

TIE will follow the demonstration-based model recommended by the GOLD guidelines (20). A respiratory nurse will provide verbal instructions using an inhaler technique education template (Attachment 3) and demonstrate manual techniques, including empty canister operations, alongside the patient. The nurse will determine when to conclude the education session based on their judgment that the patient has performed three consecutive correct attempts. Each education session will allow for ≤ 10 attempts with the empty canister.

### 2.5 Outcome measures

Table 1 outlines the items to be measured and the time window for data collection.

#### 2.5.1 Primary outcome

The primary outcome of this study will be AE frequency within 12 weeks after discharge, which will be monitored weekly via telephone.

The GOLD 2024 guidelines define AECOPD as a sudden worsening of respiratory symptoms, with dyspnea as the primary symptom. Secondary symptoms may include increased sputum volume and thickness, aggravated coughing, and wheezing. During this study's weekly follow-ups, an AE will be recorded if the patient experiences a significant worsening of dyspnea (primary symptom) lasting for ≥2 days or if two or more secondary symptoms worsen beyond normal daily variation over ≥2 days. The duration of each exacerbation will be carefully documented to prevent record duplication during subsequent follow-ups. A structured interview and record form will be developed to capture data on the number of AECOPD exacerbations over 12 weeks after discharge. See Appendix 1 for additional details.

#### 2.5.2 Secondary outcomes

The following secondary outcomes will be considered.

(1) EXACT-Pro scale score: The EXACT-Pro questionnaire allows patients to self-assess AE-related symptoms, including dyspnea, cough, sputum production, chest symptoms, and quality of life. The scale has good reliability and validity and consists of 14 items. Each item is scored from 0 to 5, with the total score being converted into a 0–100 scale based on logarithmic transformation. The higher the score, the greater the AE severity. We will collect the relevant data once daily over 12 weeks.

(2) AE severity: AE severity will be assessed according to the GOLD 2024 guidelines by using the following grading system: Mild, AE requiring short-acting bronchodilator treatment; Moderate, AE requiring short-acting bronchodilator plus antibiotics and/or oral corticosteroids treatment; and Severe, AE requiring hospitalization or emergency department visit. Based on these definitions and grading standards, a structured interview record form will be created to collect data on AE severity within 12 weeks after discharge. See Appendix 1 for additional details.

(3) All-cause mortality

(4) Inhalation technique assessment: This will be divided into inhalation flow rate evaluation and inhalation operation checklist.

(a) Inhalation flow rate evaluation: This will be performed using a portable pressure-flow measurement device (OHM-50; ORANGER DTx, Tianjin, China). While seated, the patient will use a special mouthpiece identical to the one in their regular inhalation device, as selected by the evaluator. The patient will be instructed to inhale as they normally would when taking their medication. The test will be repeated three times, and the best result will be recorded for further analysis. This evaluation will include three objective measures: PIFr, effective inspiratory time (EIT), effective inspiratory volume, and integrated inspiratory flow system (IIFS) score (IIFSs). EIT is the duration over which the inspiratory flow rate remains within the correct range during the PIFr test. EIV, representing the inhaled volume during the EIT, is determined by calculating the area under the inspiratory flow curve within the EIT. IIFS is a standardized scoring system based on objective data. IIFSs, calculated by comparing the measured inspiratory parameters with a standardized model, is a percentile-based score on a 100-point scale, reflecting the overall effectiveness of the patient's inhalation performance.

(b) Inhalation operation checklist: This will require the use of a medication-free inhaler canister equipped with an inhalation sensor for simulation. The patient will be asked to perform three simulated inhalations. The evaluator will observe the patient's inhalation steps and then record the results on the checklist by using the sensor's feedback. See Attachment 2 for the significance of the sensor feedback and the inhalation operation checklist. The entire process will be video recorded for future reference and review.

(5) Lung function: This will be assessed through spirometry using automated equipment as indicated in the guidelines.

(6) Physical fitness:

(a) 6-Min walk test: This test will be performed according to the recommendations of the European Respiratory Society/American Thoracic Society (21).

(b) Grip strength: This will be assessed bilaterally using a dynamometer (Jamar Plus+ Digital Hand Dynamometer, Fabrication Enterprises Inc, USA).

(c) Maximum voluntary contraction of knee extension: This will be measured using a handheld dynamometer (MicroFET 2; Hoggan, West Jordan, UT, USA).

(d) Respiratory muscle function: This will be assessed based on maximal inspiratory and expiratory pressures by using a Gio Digital Pressure Gauge (22).

(7) Chronic activity-related dyspnea: This will be assessed using the modified Medical Research Council dyspnea scale.

(8) Inhaler medication adherence: The Chinese version of the Test of Adherence to Inhalers (TAI) will be used to assess treatment adherence. TAI scores, ranging from 10 to 50, are used to categorize adherence into three levels: low (≤45), moderate (46–49), and high (50). This scale has good reliability and validity (23).

(9) Health-related quality of life: This will be measured using the COPD Assessment Test.

### 2.6 Data management and quality control

Patients will be required to complete the EXACT-Pro questionnaire delivered via a WeChat mini program at 9:30 a.m. daily. Research assistants will check the questionnaire completion status at 7:00 p.m. and remind patients via telephone to complete it promptly. Research assistants will also call patients weekly to inquire about their respiratory symptoms over the past week and provide verbal reminders to take their medication regularly.

To minimize measurement errors, each participant will be assessed by the same evaluator during every visit. Study data will be stored on a password-protected platform and backed up to a secure external hard drive; data access will be restricted only to authorized researchers and staff. Automatic plausibility controls will be implemented during data entry to detect any inconsistencies or inaccuracies.

### 2.7 Statistical analysis

Data analysis will follow the modified intention-to-treat principle to ensure complete randomization, minimize selection bias, and reflect real clinical practice. Missing data will be addressed using multiple imputation to reduce potential bias resulting from missing information, ensuring the robustness and accuracy of the analysis. All statistical tests will be performed according to a preestablished statistical analysis plan. SPSS (version 21.0) will be used for all data analyses, and two-tailed *p* < 0.05 will be considered to indicate statistical significance.

Continuous variables will be expressed as means ± standard deviations or median (Interquartile) where appropriate, and categorical variables as frequencies and percentages. Normality testing for continuous variables will be performed using the Shapiro–Wilk or Kolmogorov–Smirnov test, and homogeneity of variance will be assessed using the Levene test. For continuous variables that are normally distributed with equal variance, intergroup comparisons will be conducted using the independent-samples *t*-test. For continuous variables that do not meet the normality or homogeneity assumptions, the Mann–Whitney *U*-test will be employed. For discrete variables (such as the AE number and severity over 12 weeks), intergroup comparisons will be performed using the chi-square, Fisher exact, or Cochran–Mantel–Haensel χ^2^ test.

### 2.8 Sample size calculation

Our literature review indicated that the AE rate within 3 months after hospitalization for AECOPD is approximately 48.9% (5). Our preliminary data (unpublished) indicate that after VIT, the treatment failure rate at 90 days is approximately 15%. Therefore, the expected effect size between the two groups is about 33.9%. We set the significance level α = 0.05 and the statistical power at 80% (i.e., β = 0.20), with a dropout rate assumed to be 20%. The calculated values for *Z*_α_/2 and *Z*β are 1.96 and 0.84, respectively, with standard deviation σ = 0.614, effect size Δ = 0.339. Based on these parameters and sample size calculations, each group must include ≥32 patients. To ensure data accuracy and account for potential dropouts, we plan to recruit 40 patients per study group, totaling 80 participants.

### 2.9 AE reporting

Since this study will only involve nonpharmacological, low-risk inhaler technique education interventions, we do not expect serious intervention-related adverse events. The research team will closely monitor participants to collect, assess, and report any adverse events or other unintended effects actively or spontaneously reported during the interventions.

## 3 Discussion

Inhaler technique is complex. Even after receiving appropriate education, many patients may use incorrect inhalation techniques due to a lack of detailed assessment of their equipment; this may reduce the efficacy of inhaled medications. Studies thus far have primarily observed the inspiratory flow rate in patients using DPIs to assess and guide optimization of inspiratory flow rate (18); however, research on the inspiratory flow rate in patients using pMDIs or SMIs is limited. We observed that when using a pMDI or SMI, most patients exhibited excessively high PIFrs (>80 L/min), increasing medication deposition in the oropharynx and thereby hindering effective drug delivery. Studies have reported that the error rate in inhalation techniques with pMDIs is as high as 86.7%, whereas that with SMIs is 58.9%. TIE primarily involves demonstration-based teaching: a physician first demonstrates the correct technique and key points to their patient; then, the patient mimics the technique under the physician's observation and guidance. However, with this technique, errors related to hand–mouth coordination and effective inhalation duration often remain undetected.

In this study, we will use sensors to monitor inhalation duration and hand–mouth coordination dynamically. With sensor feedback, physicians can provide targeted practice to address patient errors, overcoming the limitations of traditional education methods. To the best of our knowledge, this will be the first clinical study to objectively assess the entire inhalation process and offer targeted guidance for error correction. This will also be the first study to apply a visual method for inhalation technique instruction for patients with AECOPD. Visual feedback-based educational training methods have been widely applied in rehabilitation, with successful outcomes srehabilitation (24, 25), as well as in rehabilitation for pediatric cerebral palsy (26), orthopedic (27), and pain management (28).

Here, we primarily aim to observe and compare the impacts of our short-term VIT and the TIE on AECOPD frequency within 12 weeks after discharge. We anticipate that our results will provide evidence supporting the establishment of more effective inhaler technique education processes.

## Privacy protection

This study will strictly adhere to privacy protection policies regarding the collection, sharing, and maintenance of personal information for potential participants and those enrolled in the study. All personal information will be collected only after obtaining written informed consent and will be used solely for research purposes. Data will be de-identified at the time of collection, and there will be no contractual agreements limiting researchers' access to the data set. Only authorized researchers will be allowed access to the information to ensure the confidentiality and integrity of the data. During and after the study, data sharing will be limited to situations that comply with relevant regulations, ensuring the strict protection of participants' privacy. After the study concludes, personal data will be retained for a reasonable period for review or verification, but will not be used for any unauthorized purposes and will be regularly destroyed according to legal requirements.
