# Supplementary material for: Study protocol for impact of visual inhaler technique instructions on short-term outcomes in hospitalized patients with acute exacerbation of chronic obstructive pulmonary disease
Source: Front Med (Lausanne). 2026 Jan 9;12:1735550. doi: 10.3389/fmed.2025.1735550 (PMC12827547; doi:10.3389/fmed.2025.1735550)
Supplement: Supplementary file 1 [file Data_Sheet_1.pdf]

Structured Interview Record for the Number and Severity of AECOPD Exacerbations within 12 Weeks;

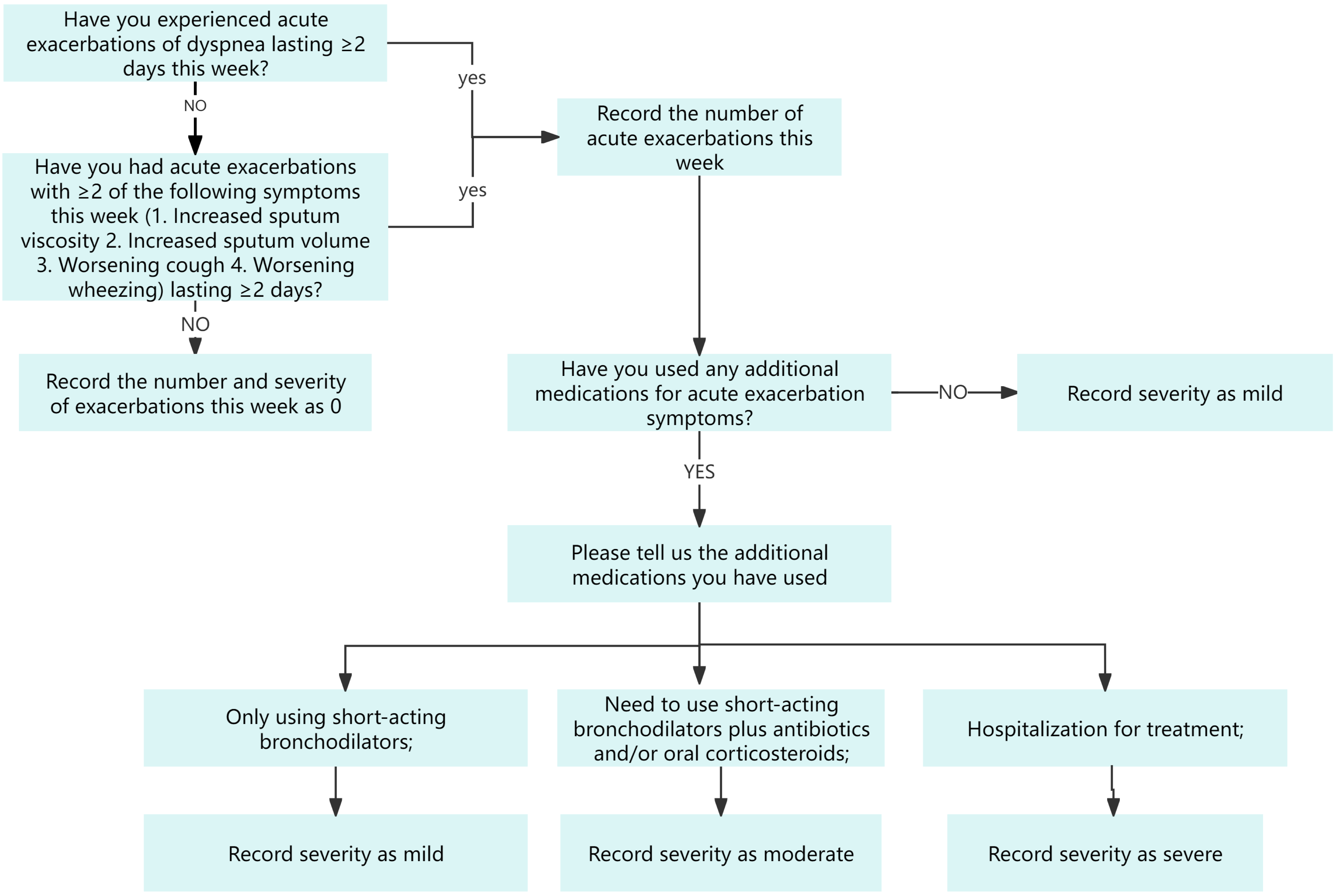

	week 1	week 2	week 3	week 4	week 5	week 6	week 7	week 8	week 9	week 10	week 11	week 12
Number of occurrences												
Severity												
