# Supplementary material for: Study protocol for impact of visual inhaler technique instructions on short-term outcomes in hospitalized patients with acute exacerbation of chronic obstructive pulmonary disease
Source: Front Med (Lausanne). 2026 Jan 9;12:1735550. doi: 10.3389/fmed.2025.1735550 (PMC12827547; doi:10.3389/fmed.2025.1735550)
Supplement: Supplementary file 2 [file Data_Sheet_2.docx]

Attachment 2

Inhaler Sensor Assembly Diagram


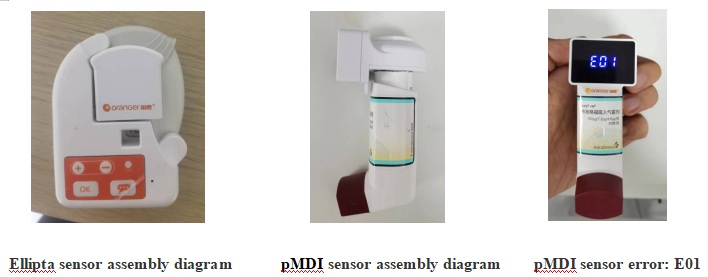


| **Checklist for inhalation operation** | | | |
| --- | --- | --- | --- |
|  | | pMDI/SMI □ | Ellipta □ |
| Remove cap | | Operational errors are unacceptable | Not applicable |
| Check dose counter | | Operational errors are unacceptable | Operational errors are unacceptable |
| Slide lever until click heard and keep the inhaler upright | | Not applicable | Operational errors are unacceptable |
| Shake pMDI well | | Operational errors are unacceptable | Not applicable |
| Breathe out normally away from the inhaler | | Operational errors reduce efficacy. | Operational errors are unacceptable |
| Hold the inhaler upright | | Operational errors reduce efficacy. | Not applicable |
| Keep head upright or slightly tilted | | Operational errors reduce efficacy. | Operational errors reduce efficacy. |
| Seal lips around mouthpiece | | Operational errors are unacceptable | Operational errors reduce efficacy. |
| Press down the inhaler to release one dose of medication while beginning to inhale slowly and deeply." | | Operational errors are unacceptable | Not applicable |
| Inhale forcefully and deeply through the mouth | | Not applicable | Operational errors are unacceptable |
| Continue slow and deep inhalation | | Operational errors are unacceptable | Not applicable |
| Hold breath for less than 5 seconds | | Operational errors are unacceptable | Operational errors are unacceptable |
| Hold breath for less than 10 seconds | | Operational errors reduce efficacy. | Operational errors reduce efficacy. |
| Breath out gently,away from your inhaler | | Not applicable | Operational errors reduce efficacy. |
| Sensor fault message | E01 | Operational errors are unacceptable | Operational errors are unacceptable |
|  | E02 | Operational errors reduce efficacy. | Operational errors reduce efficacy. |
|  | E03 | Operational errors reduce efficacy. | Operational errors reduce efficacy. |
|  | E04 | Operational errors are unacceptable | Operational errors are unacceptable |
|  | | All operations are highly standardized. | |
| pMDI/SMI(E01:No inhalation E02:Hand-breath coordination issues E03:Insufficient inhalation duration E04: Comprehensive error, indicating the presence of at least two of the above errors.  Ellipta（E01:No inhalation E02:Insufficient inhalation flow rate E03:Insufficient inhalation duration E04: Comprehensive error, indicating the presence of at least two of the above errors.） | | | |
