# Supplementary material for: Study protocol for impact of visual inhaler technique instructions on short-term outcomes in hospitalized patients with acute exacerbation of chronic obstructive pulmonary disease
Source: Front Med (Lausanne). 2026 Jan 9;12:1735550. doi: 10.3389/fmed.2025.1735550 (PMC12827547; doi:10.3389/fmed.2025.1735550)
Supplement: Supplementary file 3 [file Data_Sheet_3.docx]

Attachment 3

**pMDI/SMI Standard Inhaler Education Template:**

1. **Prepare the Inhaler**
   - Remove the mouthpiece cover.
   - Shake the inhaler vigorously for 5–10 seconds to ensure proper mixing of the medication.
   - If necessary, prime the inhaler: If it is the first use or has not been used for an extended period, spray the inhaler into the air three times to ensure it functions properly.
2. **Assume the Correct Posture**
   - Stand or sit upright to ensure optimal delivery of medication to the lungs.
3. **Exhale Completely**
   - Breathe out as fully as possible, but avoid exhaling forcefully. Ensure the exhalation is not directed toward the mouthpiece.
4. **Position the Inhaler**
   - Place the mouthpiece between your teeth and hold it firmly with your lips, ensuring a tight seal. Do not bite the mouthpiece.
5. **Inhale the Medication**
   - Begin a slow, deep inhalation while simultaneously pressing down on the canister to release one dose of medication. Continue to inhale steadily and deeply to ensure the medication reaches the lungs.
6. **Hold Your Breath**
   - After inhaling the medication, remove the inhaler from your mouth and hold your breath for 10 seconds (or as long as comfortable). This allows the medication to settle in the lungs.
7. **Resume Normal Breathing**
   - After holding your breath, exhale slowly and return to normal breathing.
8. **Repeat if Necessary**
   - If a second dose is required, wait approximately 30 seconds before repeating the steps.
9. **Complete the Process**
   - Replace the mouthpiece cover after use.
   - Rinse your mouth with water and spit out to reduce the risk of infection and irritation.

**ELLIPTA Standard Inhaler Education Template:**

1. **Check the Dose Counter**
   - The inhaler is equipped with a dose counter that decreases by one unit after each use. Ensure you are aware of the remaining doses.12
2. **Prepare the Inhaler**
   - Inspect the inhaler to ensure it is clean, dry, and undamaged.
   - Slide the mouthpiece cover gently to expose the mouthpiece. A "click" sound will indicate that the inhaler has prepared a dose of medication.
3. **Exhale Away from the Inhaler**
   - Move the inhaler away from your mouth and exhale deeply (do not exhale into the mouthpiece, as this may affect the medication’s effectiveness).
4. **Inhale the Medication**
   - Place the mouthpiece gently into your mouth. Seal your lips tightly around the mouthpiece to ensure a proper seal.
   - Inhale deeply and rapidly through the mouthpiece with a strong, steady breath. Note: The ELLIPTA is a dry powder inhaler (DPI), so the medication is inhaled via your own inspiratory effort.
   - Hold your breath for 3–4 seconds after inhaling the medication, then exhale slowly through your nose.
5. **Close the Inhaler**
   - Slide the mouthpiece cover back into place to protect the mouthpiece. The inhaler is now ready for the next use.
6. **Rinse Your Mouth**
   - Rinse your mouth three times after use to reduce the risk of oral thrush (candidiasis).
7. **Store the Inhaler**
   - Keep the inhaler in a dry place, away from moisture and extreme temperatures. Clean the mouthpiece with a dry cloth if necessary, but avoid washing it with water.
